# Spatial fidelity of MEG/EEG source estimates: A general evaluation approach

**DOI:** 10.1016/j.neuroimage.2020.117430

**Published:** 2020-10-07

**Authors:** John G. Samuelsson, Noam Peled, Fahimeh Mamashli, Jyrki Ahveninen, Matti S. Hämäläinen

**Affiliations:** a Harvard-MIT Division of Health Sciences and Technology (HST), Massachusetts Institute of Technology (MIT), Cambridge, MA 02139, USA; b Athinoula A. Martinos Center for Biomedical Imaging, Massachusetts General Hospital, Charlestown, MA 02129, USA; c Department of Radiology, Massachusetts General Hospital (MGH), Charlestown, MA 02129, USA; d Harvard Medical School, Boston, MA 02115, USA

**Keywords:** Spatial resolution, Source estimation, Patch analysis, SNR, MEG, EEG

## Abstract

Low spatial resolution is often cited as the most critical limitation of magneto- and electroencephalography (MEG and EEG), but a unifying framework for quantifying the spatial fidelity of M/EEG source estimates has yet to be established; previous studies have focused on linear estimation methods under ideal scenarios without noise. Here we present an approach that quantifies the spatial fidelity of M/EEG estimates from simulated patch activations over the entire neocortex superposed on measured resting-state data. This approach grants more generalizability in the evaluation process that allows for, e.g., comparing linear and non-linear estimates in the whole brain for different signal-to-noise ratios (SNR), number of active sources and activation waveforms. Using this framework, we evaluated the MNE, dSPM, sLORETA, eLORETA, and MxNE methods and found that the spatial fidelity varies significantly with SNR, following a largely sigmoidal curve whose shape varies depending on which aspect of spatial fidelity that is being quantified and the source estimation method. We believe that these methods and results will be useful when interpreting M/EEG source estimates as well as in methods development.

## 1. Introduction

Magneto- and electroencephalography (M/EEG) based source localization is widely utilized in basic and clinical neuroscience research, and has had critical clinical impact for applications such as preoperative functional mapping of neurosurgery patients and non-invasive localization of epileptic foci in patients suffering from drug-resistant epilepsy (Fischer et al., 2005; Gross et al., 2013; RamachandranNair et al., 2007). Although M/EEG has excellent sub-millisecond temporal resolution, precise localization of the neural activations that generate the MEG and EEG signals remains a formidable challenge and an area of intensive research (Barkley and Baumgartner, 2003; Berger, 1929; Hämäläinen et al., 1993; Hämäläinen and Lundqvist, 2019; Jerbi et al., 2004; Mosher et al., 1995). The most widely utilized distributed source estimation methods developed to date include the minimum norm estimate (MNE), the normalized MNE estimates dynamic statistical parametric mapping (dSPM) and standardized low resolution brain electromagnetic tomography (sLORETA), as well as exact low resolution brain electromagnetic tomography (eLORETA) (Dale et al., 2000; Hamalainen and Ilmoniemi, 1994; Pascual-Marqui, 2002; Pascual-Marqui et al., 2011). A relatively new non-linear source estimation method is the mixed-norm estimate (MxNE) (Gramfort et al., 2012; Ou et al., 2009). Despite this wide range of source estimation methods and the wealth of literature on the topic, quantifying their spatial fidelity which would allow for systematic performance comparisons has been difficult because there are numerous confounding factors and several different aspects of spatial fidelity that can be quantified in a variety of ways (Brookes et al., 2011; Hari and Parkkonen, 2015).

Hauk et al. (2019) summarized important resolution metrics that have been used in the past to quantify different aspects of spatial fidelity and discussed how they relate to each-other. These metrics were also quantified for MEG, EEG, as well as for combined MEG and EEG, respectively, as a function of source location for some common linear estimation methods. How spatial fidelity of *ℓ*_2_-norm operators can be enhanced by combining MEG with EEG was also studied in Molins et al. (2008). In that study, some new resolution metrics were introduced, and results were compared with a dipole fit of the N20m response during median nerve stimulation of epilepsy patients undergoing pre-surgical functional mapping. Liu et al. (2002) defined and calculated cross-talk and point spread metrics based on Monte Carlo simulations, also quantifying the added benefit of combining MEG with EEG and the impact of the number of sensors. Hauk et al. (2011) did an extensive study of MNE and its standardized versions dSPM and sLORETA for a 306-channel whole head MEG system. It was found that the choice between these methods translates into a trade-off between smaller localization error (sLORETA) and smaller spatial dispersion (MNE). It was also noted that parameters that provide intuitive measures of spatial fidelity for easy comparisons of source estimation methods are warranted but should be interpreted with caution since much information is lost in the transformation from a distribution to a single scalar value. These studies assumed point sources and specifically investigated linear estimation methods under ideal scenarios without noise. Under these conditions, closed-form expressions of the resolution matrix are attainable. Babiloni et al. (2004) conducted a simulation study that also assessed the added benefit of integrating MEG with EEG for varying SNR but spatial fidelity was only evaluated for five selected regions of interest in the brain.

In this study, we present an approach that can be used to quantify the spatial fidelity of both linear and non-linear source estimation methods in the whole brain and, critically, how it varies as a function of SNR and activation patterns. Using this approach, we evaluate MNE, dSPM, sLORETA, eLORETA, and MxNE, with a particular emphasis on studying the effects of SNR.

While a more detailed description of the investigated source estimation methods is given in the Methods section, we include a brief summary here. MNE estimates sources by finding the source distribution that minimizes the sum of the difference between the measured MEG data and those generated by the source estimate and a regularization term, which is proportional to the *ℓ*_2_-norm of the source estimate. MNE is linear because the solution to this minimization problem can be written as a product of the sensor data and a matrix called the inverse operator or imaging kernel. dSPM and sLORETA are derived from MNE but here the imaging kernel (the same as in MNE) is multiplied by a diagonal weighting matrix, which is different in dSPM and sLORETA. eLORETA is also a linear estimate but in this case the imaging kernel is not the same as in MNE, instead it is found by iteratively solving an equation which results in minimized localization error for noise-free data. The mixed-norm estimate (MxNE) minimizes a mixed *ℓ*_2_-*ℓ*_1_ norm of the currents over time and space in the regularization term. The solution to this minimization problem cannot be written as a data-invariant imaging kernel multiplied by the sensor data. Therefore, MxNE is a non-linear source estimation method.

Because earlier work on systematic evaluation of estimation methods have relied on the analytical resolution matrix, a systematic comparison between linear and non-linear estimates has not been possible. This lack of a generally applicable evaluation method is getting even more critical with the introduction of deep learning based source estimators which are generally highly non-linear (Dinh et al., 2019). The present work aims to bridge this gap by introducing a general framework for systematic comparisons of all distributed source estimates.

## 2. Methods

The software used in this study was implemented in Python 3.7 using the MNE-Python version 0.18 software package (Gramfort et al., 2013a) as a foundation.

### 2.1. Data acquisition

MEG and EEG resting-state and structural MRI data were collected after obtaining an informed consent from healthy subjects (ages 22, 24, 31, 32 and 34, two females and three males) under a protocol approved by the Massachusetts General Hospital Institutional Review Board. T1-weighted anatomical images were obtained using a multi-echo Magnetization Prepared Rapid Gradient Echo (MEMPRAGE) (van der Kouwe et al., 2008) pulse sequence (TR = 2530 ms; 4 echoes with TEs = 1.69, 3.55, 5.41, 7.27 ms; 176 sagittal slices with 1 × 1 × 1 mm^3^ voxels, 256 × 256 mm^2^ matrix; flip angle = 7°) in a 3T Siemens Prisma whole-body MRI scanner (Siemens Medical Systems, Erlangen, Germany) using a 64 channel head and neck coil. A FLASH sequence was used to obtain the inner skull, outer skull, and scalp compartments needed for MEG/EEG forward modeling. The employed MEG system was an Elekta-Neuromag VectorView (Megin Oy, Helsinki, Finland) with 306 channels arranged in 102 triplets of one magnetometer and two orthogonal planar gradiometers. The EEG was recorded with the EEG system integrated with the MEG system using a 70-channel MEG-compatible EEG cap (EasyCap GmbH, Germany). Two electrodes for measuring electrocardiogram (ECG) were placed over the subject’s chest and two electrode pairs for measuring horizontal and vertical electrooculogram (EOG) were placed near the subject’s eyes. The resting-state data were recorded in a quiet, magnetically shielded room (IMEDCO AG, Switzerland) while the subjects sat down, relaxed, and kept their eyes open. MEG and EEG signals were bandpass filtered to 0.03 – 330 Hz and digitized at 1000 Hz sampling frequency.

### 2.2. Data preprocessing

MEG data were Maxwell filtered in MNE-python (Taulu and Kajola, 2005; Taulu and Simola, 2006) and the bad MEG sensors were replaced using the Maxwell filtering interpolation function. The bad EEG channels were excluded from further analysis. The ECG and EOG signals were used to find cardiac and ocular events which were projected from the data using signal subspace projection (SSP) (Uusitalo and Ilmoniemi, 1997). Zero to two projection vectors were applied for ocular and cardiac events, determined on a case-by-case basis. The raw resting state recordings were then cropped to include only the first 68 seconds. The recording segment was divided up into 135 epochs, each 0.5 seconds long. Evoked responses were simulated by superposing a simulated signal lasting 100 milliseconds with the average of 49 resting state epochs, randomly drawn from the available 135 epochs. The remaining 86 epochs were used to compute the noise covariance matrix that was used in source estimation.

### 2.3. Source space and forward model

The structural MRI data were preprocessed using FreeSurfer (Dale et al., 1999; Fischl et al., 1999). Cortical surfaces were tessellated into triangular meshes with ~130,000 vertices in each hemisphere. The inflated surfaces computed by FreeSurfer were used to expose the sulci in the visualization of cortical data. The cortical visualizations were made using Multi-Modal Neuroimaging Analysis and Visualization Tool (MMVT) (Felsenstein et al., 2019). The dense triangulation of the folded cortical surface provided by FreeSurfer was decimated to a grid of ~10,000 candidate source locations per hemisphere (~20,000 total), corresponding to a spacing of approximately 3.1 mm between adjacent source locations. The orientations of the dipole sources were fixed to be normal to the cortical surface throughout this study (fixed orientation constraints). A piecewise homogenous conductor model with three compartments bounded by the inner skull, outer skull, and outer skin and electrical conductivities 0.3, 0.006 and 0.3 S/m for the brain, skull, and scalp, respectively, was employed. The boundary element method (BEM) was used to compute the gain matrix (Hamalainen and Sarvas, 1989; Mosher et al., 1999). The tessellations contained 2562 vertices on each of the boundary surfaces and the linear collocation method was used in BEM. The locations and orientations of the EEG electrodes and MEG sensors with respect to the head were adopted from the experimental data.

### 2.4. Source model parcellation

**Fig. 1.**
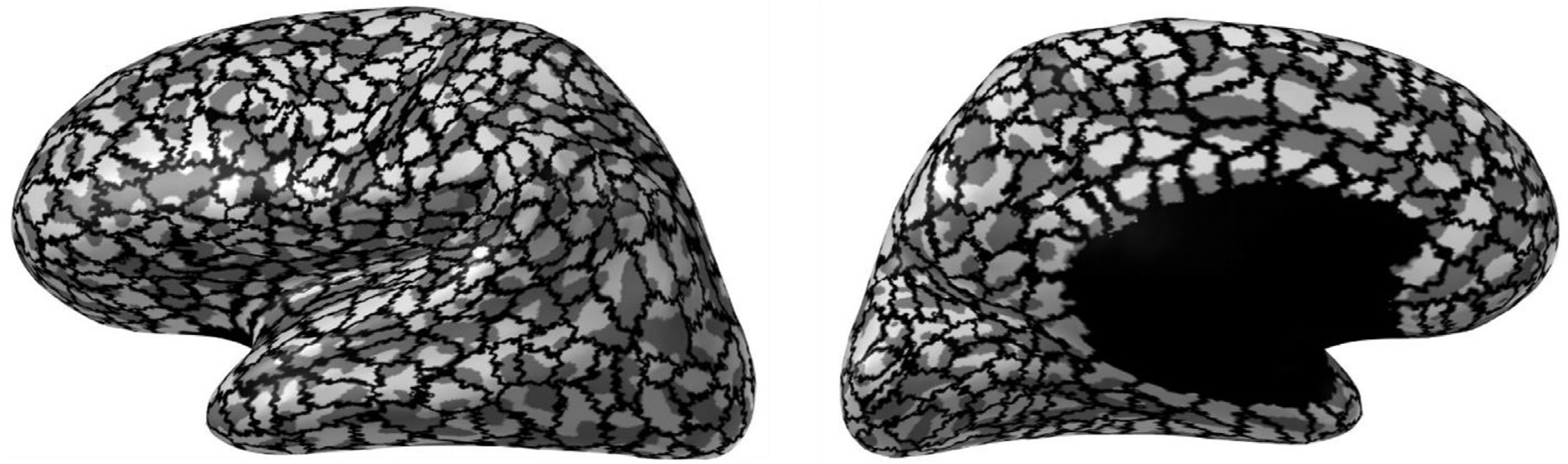
Source space parcellation. Each hemisphere is divided up into 500 non-overlapping patches, which are activated individually and uniformly in our simulations. Labels that are colored black (corpus callosum and deep brain) were excluded. The cortex has been inflated for illustrative purposes.

The Lausanne parcellation (Fig. 1) was used to group the cerebral cortex of each hemisphere into 502 different brain regions covering the entirety of the cortex (Daducci et al., 2012). The inferior part of the medial surface marked as “unknown ” in the parcellation atlas was excluded, since this region does not represent an actual surface or structure, as was the corpus callosum, which is a white matter structure, leaving a total of 1000 disjoint cortical patches. The parcellation was done by morphing brain labels from a template to the individual subject. To avoid overlap between patches, a morphing map was created between the vertices of the individual subject’s cortical surface mesh and the vertices of the template brain. Using the morphing map, each vertex in the individual subject mesh *v*_ind_ was assigned to a corresponding group of vertices *v*_template_ in the template mesh. Because each vertex in *v*_template_ was associated with a cortical label (patch) and each vertex in *v*_ind_ was associated with a number of vertices in *v*_template_, each vertex in *v*_ind_ could be associated with a number of cortical labels. Each vertex in *v*_ind_ was then categorized as belonging to that cortical label with which it had the greatest number of connections. The vertices in the individual source space *v*_*ind*_ were thus disjointly grouped into cortical labels, in accordance with the Lausanne parcellation. The number of current dipole sources per cortical label varied from 4 to 65 with a median of 17. The areas of the patches varied from 47 mm^2^ to 722 mm^2^ (mean = 253 mm^2^, std = 117 mm^2^).

### 2.5. Empirical resolution matrix

We first introduced the concept of an empirical resolution matrix in Krishnaswamy et al. (2017); in the present study we developed this concept further. In the following, we first describe how the empirical resolution matrix is found in each subject, then the implementation of the tested inverse methods, and finally the analytical metrics that were used to evaluate the spatial fidelity of the inverse methods. Throughout this paper, scalar variables are denoted by lowercase characters, vectors by boldface lowercase characters, and matrices by boldface uppercase characters.

The M/EEG sensor space signal ***y*** resulting from an active source distribution ***x*** can be computed at any given time point *t* by the gain matrix ***G***;

$$y_{t}=Gx_{t}.$$

For linear inverse methods, the inverse solution ${\hat{x}}_{t}$ is found by multiplying the recordings with an inverse kernel ***K***;

(1) $${\hat{x}}_{t}=Ky_{t}$$

hence

(2) $${\hat{x}}_{t}=KGx_{t}=Rx_{t},$$

where **R** = **KG** is called the *resolution matrix*. Under ideal circumstances, the source estimate ${\hat{x}}_{t}$ would be equal to the actual source ***x***_*t*_, so that **R** = **I**. Since the resolution matrix links the source estimate ${\hat{x}}_{t}$ to the actual source ***x***_*t*_, it provides insight into the performance of the source estimation method. However, this closed-form expression of the resolution matrix used in Eq. 2 is only attainable when the inverse method is linear, i.e., when the relationship between the signal in the sensors ***y***_*t*_ and the source estimation ${\hat{x}}_{t}$ can be written as a linear transformation ***K*** as in Eq. 1. Furthermore, it cannot be used to evaluate an inverse method’s robustness to noise. Because of these limitations, we instead simulate the signal and superpose noise,

Y=GX+N,

where ***Y*** is the sensor data matrix accumulated over time with dimensions *n*_*r*_ × *n*_*t*_ (*n*_*r*_ is the number of sensors and *n*_*t*_ is the number of time points), ***X*** is the source activation matrix of dimensions *n*_*s*_ × *n*_*t*_ (*n*_*s*_ is the number of sources) and ***N*** is the noise matrix of dimensions *n*_*r*_ × *n*_*t*_. Estimating the source of this sensor data matrix gives us the source estimation matrix $\hat{X}$;

$$\hat{X}=f(Y)=f(GX+N),$$

where *f* is the inverse operator, which is not necessarily linear. The performance of some spatiotemporal signal processing or source estimation methods, e.g., TF-mixed-norm, C-MNE, CSS and Kalman filter approaches, varies with the activation waveforms (Dinh et al., 2019; Gramfort et al., 2013b; Lamus et al., 2012; Pirondini et al., 2018; Samuelsson et al., 2019). This variability can be examined by varying the activation waveforms ***X***, e.g., in terms of the number of active sources or degree of correlation between the sources. Furthermore, robustness to noise can be tested by varying ***N***.

In this study, we simulated activation of one cortical patch at a time, each activation lasting 100 milliseconds with constant source dipole amplitude oriented normally to the patch surface. The cortical patches were delineated by the brain labels in the Lausanne parcellation (Fig. 1). The patches were uniformly activated and the average size of 2.5 cm^2^ corresponds roughly to the expected spatial extent of the synchronously active neuronal assemblies that can be observed non-invasively with M/EEG (Murakami and Okada, 2015).

**Fig. 2.**
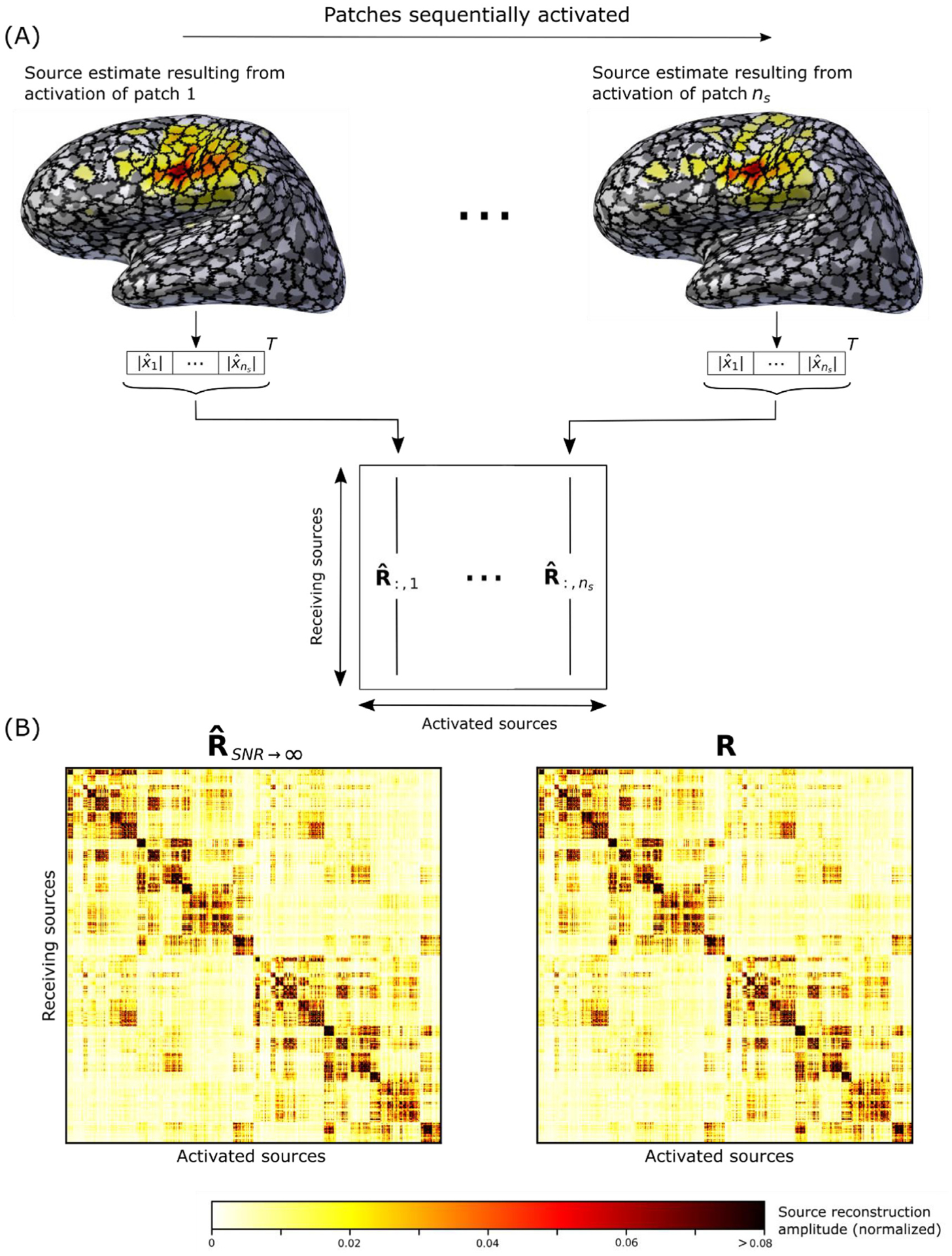
A) Conceptual schematic of the empirical resolution matrix $\hat{R}$. Each entry ${\hat{R}}_{ij}$ is the average of all source dipole amplitudes in patch *i* over time during activation of patch *j* (Eq. 3). B) Empirical resolution matrix $\hat{R}$ for the limit when SNR goes to infinity (left) which converge to the analytical resolution matrix ***R*** (right). The displayed resolution matrices are for MNE in one subject.

By simulating activation of each patch individually and then performing a source reconstruction of the resulting sensor space signal superposed with noise, an empirical resolution matrix ${\hat{R}}_{t}$ can be generated by populating each column of ${\hat{R}}_{t}$ with the amplitude of the resulting source estimate ${\hat{x}}_{t}$, as outlined in Fig. 2. In this study we will be using the average of ${\hat{R}}_{t}$ over time, denoted $\hat{R}$, which is of dimensions (*n*_*s*_, *n*_*s*_), where *n*_*s*_ = 1000. The entry ${\hat{R}}_{i,j}$ is thus the average of all dipole source amplitudes in cortical patch *i* over time resulting from activation of patch *j*;

(3) $${\hat{R}}_{i,j}=\frac{\sum_{t=1}^{n_{t}}\sum_{k\in P_{i}}\left|{\hat{X}}_{k,t}\right|}{n_{t}\left|P_{i}\right|},$$

$$X_{k,t}=\{\begin{array}{c} 10\text{ nAmif }k\in P_{j}\\ 0\text{if }k\notin P_{j} \end{array},$$

where *P*_*i*_ is the set of source dipoles in patch *i*. The SNR is then adjusted by scaling the signal by a dimensionless parameter *α*;

Y=αGX+N,

$$\alpha =\frac{\text{SNR}}{\Sigma_{j=1}^{3}\left(\frac{\sum_{i=1}^{n_{s}}{\left\|G_{{\left\{\cdot \right\}}_{j},i}x_{i,:}\right\|}_{E}}{3n_{S}{\left\|N_{{\left\{\cdot \right\}}_{j},:}\right\|}_{E}}\right)},$$

where the ‖‖_*E*_ operator denotes the mean signal energy across sensors, {·}_*j*=1,2,3_ indicates the sets of sensor indices corresponding to magnetometers, gradiometers and EEG, respectively, *i* corresponds to the source indicies of different patches and *n*_*s*_ is, as before, the number of patches (*n*_*s*_ = 1000). Thus, we scale the signal so that the mean SNR across patch activation and modalities is equal to the predetermined SNR. This is important because magnetometers, gradiometers and EEG have different sensitivities for a given source and this difference in sensitivity also varies over different sources (Goldenholz et al., 2009; Hunold et al., 2016; Samuelsson et al., 2019). By scaling the signal so that the average SNR over patch activations and modalities is equal to the predetermined SNR, we can examine a source estimation method’s performance over the whole brain for a given SNR without changing the activation amplitudes of the different patches. Importantly, changing the activation amplitudes is not physiologically realistic and obscures comparisons to earlier studies and the spatial variability of the performance of the estimator. When SNR is infinite, the noise term is zero and the empirical resolution matrix $\hat{R}$ converges to the closed-form resolution matrix ***R*** when using a linear source estimation method, e.g., MNE (Fig. 2 b). Since ***R*** is easily accessible, this was used as a verification of our simulations. The initial approach that was introduced in Krishnaswamy et al. (2017) used point sources instead of cortical patch activations without any noise added to the sensor data.

### 2.6. Inverse methods

We evaluated MNE, eLORETA, dSPM and sLORETA, as well as the non-linear *ℓ*_1_-*ℓ*_2_ mixed norm estimate (MxNE) (Dale et al., 2000; Gramfort et al., 2012; Hamalainen and Ilmoniemi, 1994; Ou et al., 2009; Pascual-Marqui, 2002; Pascual-Marqui et al., 2011). All methods were implemented using surface-constrained fixed orientation dipoles without depth-bias compensation.

In MNE, the source estimate ${\hat{x}}_{t}$ at time *t* is found by solving the optimization problem,

(4) $${\hat{x}}_{t}=\underset{x_{t}}{\text{argmin}}\left({\left\|y_{t}-Gx_{t}\right\|}_{C}^{2}+\lambda^{2}{\left\|x_{t}\right\|}^{2}\right)$$

where the notation = ‖**a**‖_*w*_ = **a**^*T*^**W**^−1^ **a** is the **W**^−1^ -weighted norm of ***a***, ***x***_*t*_ are as before the active sources, ***y***_*t*_ are the sensor data, ***G*** is the gain matrix, ***C*** is the noise covariance matrix and *λ* is the Tikhonov regularization parameter. Solving this optimization problem yields a linear relationship between the sensor data ***y***_*t*_ and source estimate ${\hat{x}}_{t}$ (Eq. 1). In the case of MNE, the inverse Kernel ***K*** that relates the recorded data ***y***_*t*_ to the source estimate ${\hat{x}}_{t}$ can be written in closed form as

$$K_{\text{MNE}}=G^{T}{\left(GG^{T}+\lambda^{2}C\right)}^{-1}.$$

Although the MNE solution is the most straightforward source estimate, it has a few undesirable properties, e.g., mislocalization of deeper sources to more superficial locations. Attempts have been made to address these issues, e.g., dSPM and sLORETA, by standardization of the MNE solution by a diagonal weighting matrix ***W***;

$$K^{*}=WK_{\text{MNE}}.$$

What separates dSPM from sLORETA is that the weights ***W*** are different (Hauk et al., 2011). In dSPM, each source is divided by the energy of the noise mapped to that location by using the noise covariance matrix, so that

$$W_{{\text{dSPM}}_{i,i}}^{2}=\frac{1}{{\text{diag}}_{i}\left(K_{\text{MNE}}CK_{\text{MNE}}^{T}\right)}.$$

In sLORETA, the weights are the inverses of the diagonal entries of the MNE resolution matrix;

$$W_{{\text{sLORETA }}_{i,i}}^{2}=\frac{1}{{\text{diag}}_{i}\left(K_{\text{MNE}}G\right)}=\frac{1}{{\text{diag}}_{i}\left(G^{T}{\left(GG^{T}+\lambda^{2}C\right)}^{-1}G\right)}\;=\frac{1}{{\text{diag}}_{i}\left(K_{\text{MNE}}\left(GG^{T}+\lambda^{2}C\right)K_{\text{MNE}}^{T}\right)}.$$

The eLORETA (Pascual-Marqui et al., 2011) estimation also corresponds to a linear operator;

$$K_{\text{eLORETA}}=D^{-1}G^{T}{\left(GD^{-1}G^{T}+\lambda^{2}C\right)}^{-1},$$

where ***D*** is a diagonal matrix with elements *d*_*i*_ found by iteratively solving the nonlinear system of equations

$$d_{i}=\frac{1}{x_{0}}\sqrt{G_{:,i}^{T}{\left(GD^{-1}G^{T}+\lambda^{2}C\right)}^{-1}G_{:,i}},$$

where *x*_0_ = 1 Am is the unit current amplitude. This constant was not included in the original eLORETA publication but is necessary to maintain the correct unit [**D**] = 1∕Am^2^, and follows directly from a closer look at the derivation of eLORETA. The eLORETA approach can be thought of as seeking an optimal depth weighting (Lin et al., 2006) resulting in minimized localization errors for noise-free data. Thus, MNE, dSPM, sLORETA and eLORETA are all linear estimation methods because they can be written in the form outlined in Eq. 1. MNE and eLORETA result in solutions in physical current units (Am) whereas dSPM and sLORETA transform the data into dimensionless statistical quantities. The mixed-norm estimate is non-linear because it uses a mixed *ℓ*_1_-*ℓ*_2_ norm instead of the *ℓ*_2_-norm in the regularization term in Eq. 4., and its source estimate $\hat{x}$ can consequently not be written in the form of Eq. 1. Instead, the optimization problem is solved by an iterative gradient-based approach (Gramfort et al., 2012; Strohmeier et al., 2014).

In the present study, the regularization parameter *λ* was set to *λ*^2^ = SNR^−2^ in the linear methods. Since the noise term was scaled to a pre-determined SNR, the SNR was well-defined and did not have to be estimated. In the mixed norm estimates, the regularization parameter *α* was held constant at 55, which is a compromise between no regularization (*α*=0) and full regularization (*α*=100) which gives zero source activity estimates. The iterative optimization procedure that is used in MxNE was performed with a tolerance of 10^−6^ and the maximum number of iterations was 3000, which are the default values.

Another common source estimation technique is beamforming, which we in the present study do not include in our analysis. The difficulty of assessing beamformers in the same framework is that they are sensitive to forward modeling errors (Steinsträter et al., 2010) and usually rely on a volumetric rather than a surface source space that is commonly used in distributed estimates. Comparing these methods therefore also amounts to disentangling the effects of employing a volumetric or surface source space. Because we simulate activity from cortical patch activations and then use the same surface source space in the inverse problem for the distributed source estimators, these estimators would have an obvious advantage over the ones employing a volumetric source space which would make performance assessment biased and is why we excluded beamformers from the present study.

### 2.7. Spatial resolution metrics

Although much information is lost when transforming a resolution matrix into scalar numbers, these numbers can still be useful when chosen so that they provide a comprehensive description of the source estimates. To that end, there are three aspects of source estimation performance that should be considered; localization accuracy, spatial dispersion (blurriness) and amplitude reconstruction (Hauk et al., 2019). In the present study we will focus on localization accuracy and spatial dispersion because comparing estimation methods that result in different units such as MNE and dSPM makes amplitude reconstruction performance difficult to assess in a general way.

We quantify localization error PE by the Euclidean distance in 3D space between the active source *r*_*i*_ and the position of the peak source reconstruction amplitude ***r***_*j*_;

$$\text{PE}={\left\|r_{i}-r_{j}\right\|}_{2},$$

$$j=\underset{k}{\text{argmax}}{\left\{\left|{\hat{x}}_{k}\right|\right\}}_{k=1,\ldots ,N_{s}},$$

where *N*_*s*_ is the number of source dipoles that is not to be confused with *n*_*s*_ which is the number of patches. Because we study patch activations, the point source vertex *i* that defines the center of the patch is determined by the point source in the patch that is closest to the center of gravity of the patch in the spherically inflated surface of the hemisphere;

$$i=\underset{k}{\text{argmin}}\left({\left\{{\left\|r_{cg}^{\prime }-r_{k}^{\prime }\right\|}_{2}\right\}}_{k\in P}\right),$$

where $r_{k}^{\prime }$ denotes the position of vertex *k* on the spherical surface, *P* is the set of source dipoles in the activated patch and

$$r_{cg}^{\prime }=\frac{\sum_{k\in P}r_{k}^{\prime }}{\left|P\right|}$$

is the center of gravity, or average, of all source positions in the activated patch in the spherically inflated surface space.

We quantify spatial dispersion SD as

$$\text{SD}=\frac{\sum_{k=1}^{N_{s}}d_{jk}\left|{\hat{x}}_{k}\right|}{\sum_{k=1}^{N_{s}}\left|{\hat{x}}_{k}\right|},d_{jk}={\left\|r_{j}-r_{k}\right\|}_{2},$$

which is the *ℓ*_1_ norm of the distance vector $d={\left\{d_{jk}\right\}}_{k=1,\ldots ,N_{s}}$, containing the distances *d*_*jk*_ between the position of the peak reconstruction amplitude ***r***_*j*_ and all sources in the source space ${\left\{r_{k}\right\}}_{k=1,\ldots ,N_{s}}$, weighted by the source estimate $\hat{x}$ normalized to its *ℓ*_1_ norm. We chose the *ℓ*_1_ norm instead of the *ℓ*_2_ norm to mitigate the effect of outliers.

**Fig. 3.**
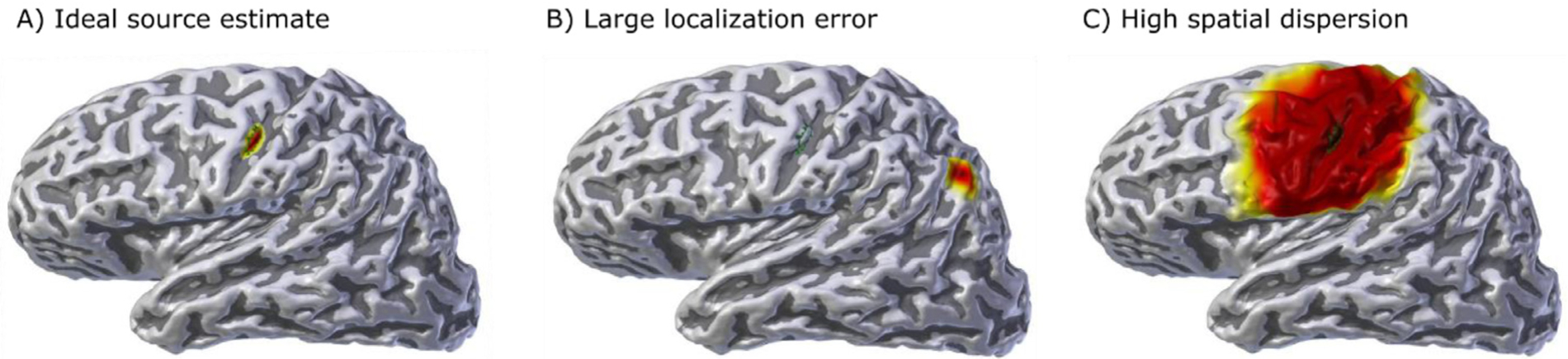
Visualization of spatial resolution metrics, the green contour outlines the activated patch: a) ideal source estimate; b) source estimate with large localization error (quantified as high PE); c) source estimate with high spatial dispersion (high SD).

In an ideal scenario, SD and PE are both zero for all patches on the cortical surface. Fig. 3 displays a visualization of what these metrics quantify.

### 2.8. Receiver-operator and precision-recall characteristics (ROC/PRC)

While the resolution metrics defined above only apply when one source is active at a time, a more generalized evaluation approach that does not assume single source activation is possible by using receiver operator characteristics (ROC) (Brown and Davis, 2006). ROC analysis quantifies the performance of a binary classifier system as its discrimination threshold *T* is varied. Here, the normalized reconstructed source patches $\tilde{\hat{x}}$ *i* are classified as active/not active if the mean source reconstruction amplitude in the patch is greater/smaller than the threshold *T*;

$$f\left({\tilde{\hat{x}}}_{i},T\right)=\{\begin{array}{cc} 1\;(\text{ active }) & \text{ if }{\tilde{\hat{x}}}_{i}>T\\ 0\text{ (not active) } & \text{ if }{\tilde{\hat{x}}}_{i}<T, \end{array}$$

$${\tilde{\hat{x}}}_{i}=\frac{{\hat{x}}_{i}}{\max\left({\left\{{\hat{x}}_{i}\right\}}_{i=1,\ldots ,n_{s}}\right)}|x=\delta_{j},$$

$${\hat{x}}_{i}=\frac{\sum_{t=1}^{n_{t}}\sum_{k\in P_{i}}\left|{\hat{X}}_{k,t}\right|}{n_{t}\left|P_{i}\right|}={\hat{R}}_{i,j},$$

$$\Rightarrow f\left({\tilde{\hat{R}}}_{ij},T\right)=F_{T},$$

where ***F***_*T*_ is a binary matrix of dimensions (*n*_*s*_, *n*_*s*_) and ${\tilde{\hat{R}}}_{ij}$ is the resolution matrix that has been normalized to the maximum value of each column;

$${\tilde{\hat{R}}}_{:,j}=\frac{{\hat{R}}_{:,j}}{\text{max}\left({\hat{R}}_{:,j}\right)}.$$

Ideally, ***F***_*T*_ would be the identity matrix for all values of *T* in the interval 0 *< T <* 1. Since the active sources are the diagonal entries of ***F***_*T*_ and the non-active sources are the off-diagonal entries, ***F***_*T*_ can be turned into the number of true positives (TP) by summing all diagonal entries, true negatives (TN) by summing all off-diagonal entries that are zero, false positives (FP) by summing all off-diagonal entries and false negatives (FN) by summing all diagonal entries that are zero;

$$\text{TP}=\sum_{i=j}F_{Ti,j},$$

$$\text{FP}=\sum_{i\neq j}F_{Ti,j},$$

$$\text{TN}=\sum_{i\neq j}\delta \left(F_{Ti,j},0\right),$$

$$\text{FN}=\sum_{i=j}\delta \left(F_{Ti,j},0\right),$$

where

$$\delta \left(F_{Ti,j},0\right)=\{\begin{array}{l} 1\;if\;F_{Ti,j}=0\\ 0\;if\;F_{Ti,j}\neq 0 \end{array}.$$

The true positive rate (TPR) and false positive rate (FPR) can then be calculated as:

$$\text{TPR}=\frac{\text{TP}}{\text{TP}+\text{FN}},\text{FPR}=\frac{\text{FP}}{\text{FP}+\text{TN}}.$$

Precision-recall characteristics (PRC) is another way of assessing performance and has been suggested to be a better metric of success on imbalanced data sets such as the present one where we have 999000 negatives and 1000 positives (Saito and Rehmsmeier, 2015). Here, positive predictive value (PPV) or “precision ”, is on the y-axis and defined as

$$\text{PPV}=\frac{\text{TP}}{\text{TP}+\text{FP}},$$

and TPR as defined above, or sensitivity, is on the x-axis. As the threshold *T* → 1, the number of positives tend to zero and TPR goes to zero but PPV is undefined (zero divided by zero). In this limit, we replace the undefined PPV with the PPV for the highest threshold below 1, presuming a horizontal asymptote. The combination of an empirical resolution matrix $\hat{R}$ and a threshold value *T* can thus be transformed into one point on the (FPR, TPR)- or (TPR, PPV)-plane. An ROC/PRC curve is then generated by varying *T*. Integrating the area under the ROC curve (AUROC) or PRC curve (AUPRC) gives a value between 0 (always wrong) to 1 (always right). For AUROC, 0.5 is the expected result from random guessing. AUC (ROC/PRC) can then be used as a metric for spatial fidelity of source estimation methods.

## 3. Results

### 3.1. Resolution matrix

**Fig. 4.**
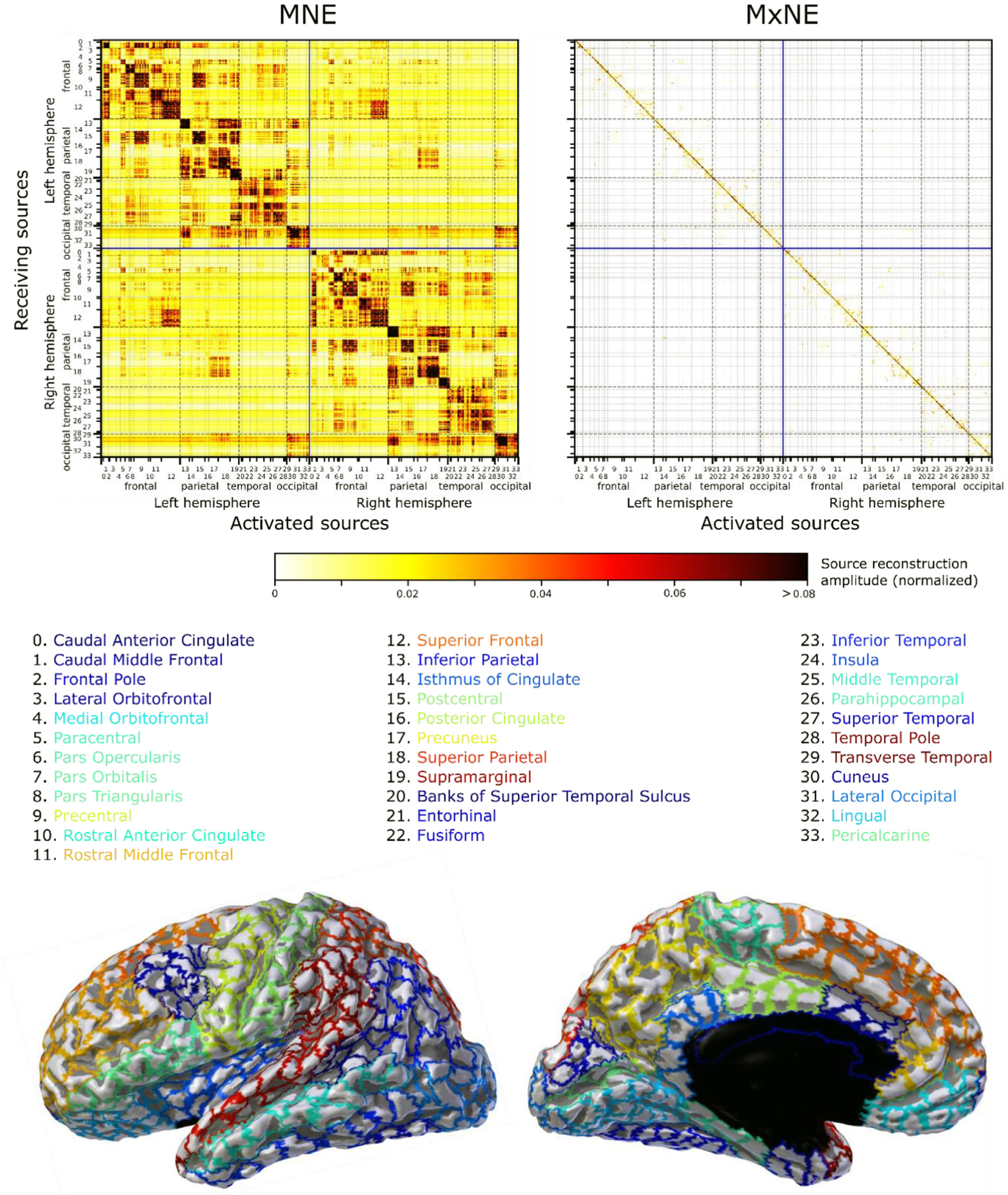
Empirical resolution matrix $\hat{R}$ averaged over all subjects for SNR = 3, normalized to the largest value in the matrix. Sources have been grouped together into 34 brain regions, marked as numbers with a legend below that is color coded with the parcellation shown at the bottom. These brain regions are delineated by the minor grids and are in turn grouped together into lobes which are delineated by major grids. The lobes are divided into left and right hemispheres, marked by the blue lines.

Fig. 4 shows the empirical resolution matrix $\hat{R}$ averaged over subjects for MNE and MxNE, SNR = 3. While MNE has a continuum of source activation amplitudes, the majority of non-active sources are estimated to be zero with MxNE. The sparsity of the MxNE estimates is thus immediately apparent in Fig. 4. The clearest general trend is that proximal sources share the highest point spread and cross talk; a three-tiered block-diagonal structure is present in the resolution matrix of MNE, as well as other linear methods, with the most significant cross-talk happening between sources in the same brain region, delineated by the minor grids in Fig. 4. The second most significant cross-talk is between sources in the same lobe, delineated by major grids, and then between sources in the same hemisphere. This structure is less apparent in MxNE due to its very sparse estimates. There is, however, quite significant inter-hemispheric cross-talk in MNE between sources on the medial surface, e.g., between the right and left superior frontal gyrus and between the right and left cuneus.

### 3.2. Resolution metrics

**Fig. 5.**
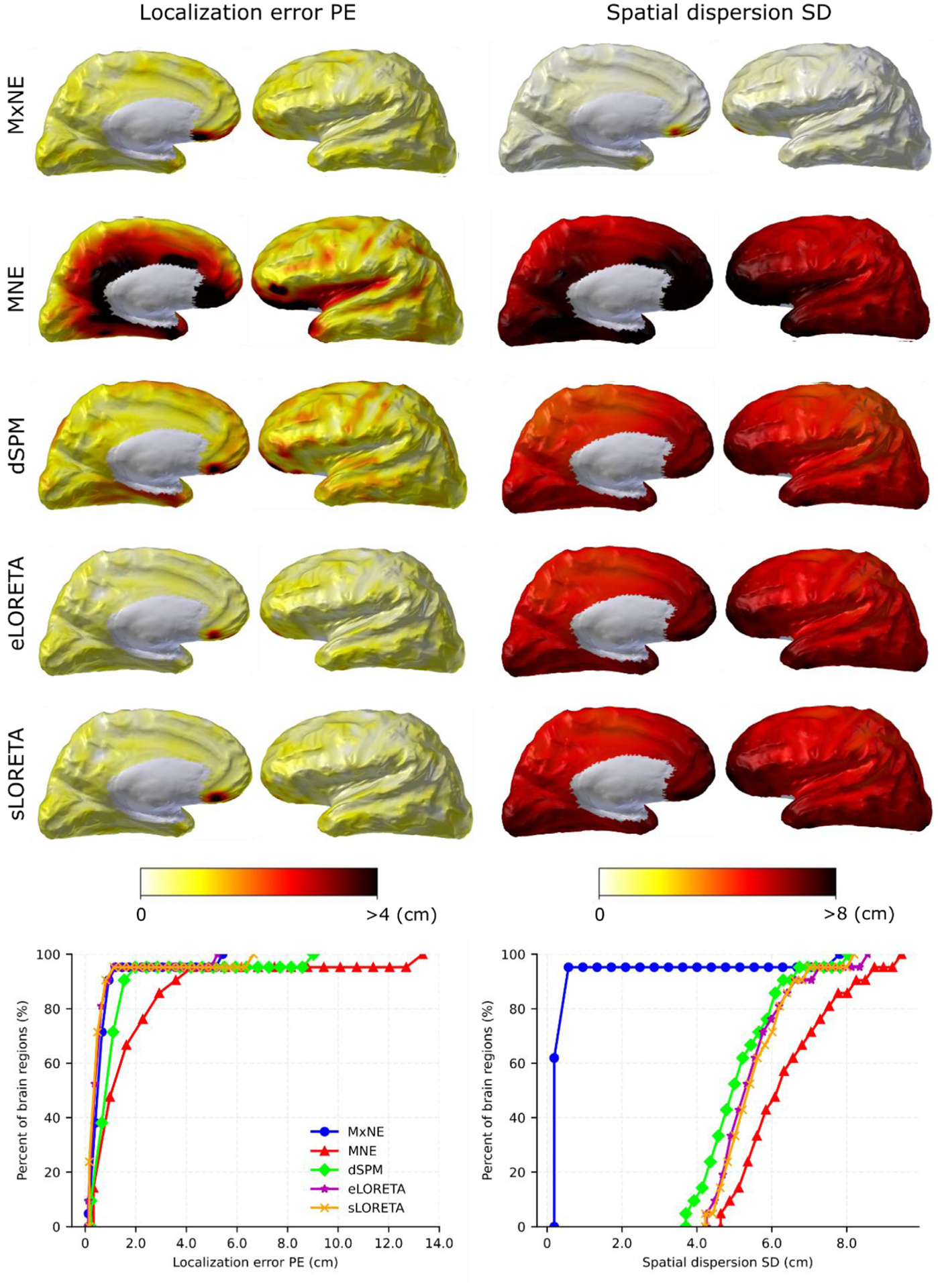
Localization error PE (left) and spatial dispersion SD (right) for different methods (rows) and SNR = 3, averaged over subjects. Represented in topographic plots and cumulative histograms. The cerebral hemispheres have been partly inflated for illustrative purposes (inflation = 0.8).

Fig. 5 shows the localization error PE and spatial dispersion SD of all sources for SNR = 3. These resolution data are displayed both as a function of their location represented as topographic plots over the cortical mantle and as cumulative histograms. The overall difference between the linear methods is relatively small both in terms of localization error and spatial dispersion although s/eLORETA have lower localization errors at this SNR (= 3) than dSPM and MNE while dSPM has somewhat lower spatial dispersion. The non-linear MxNE has significantly lower spatial dispersion and a localization error as low as s/eLORETA. The cumulative plots in the lower end of Fig. 5 shows that the peak source reconstruction amplitude rarely coincides exactly with the center of the patch, including for s/eLORETA, which otherwise exhibit zero localization error for point sources and infinite SNR.

**Fig. 6.**
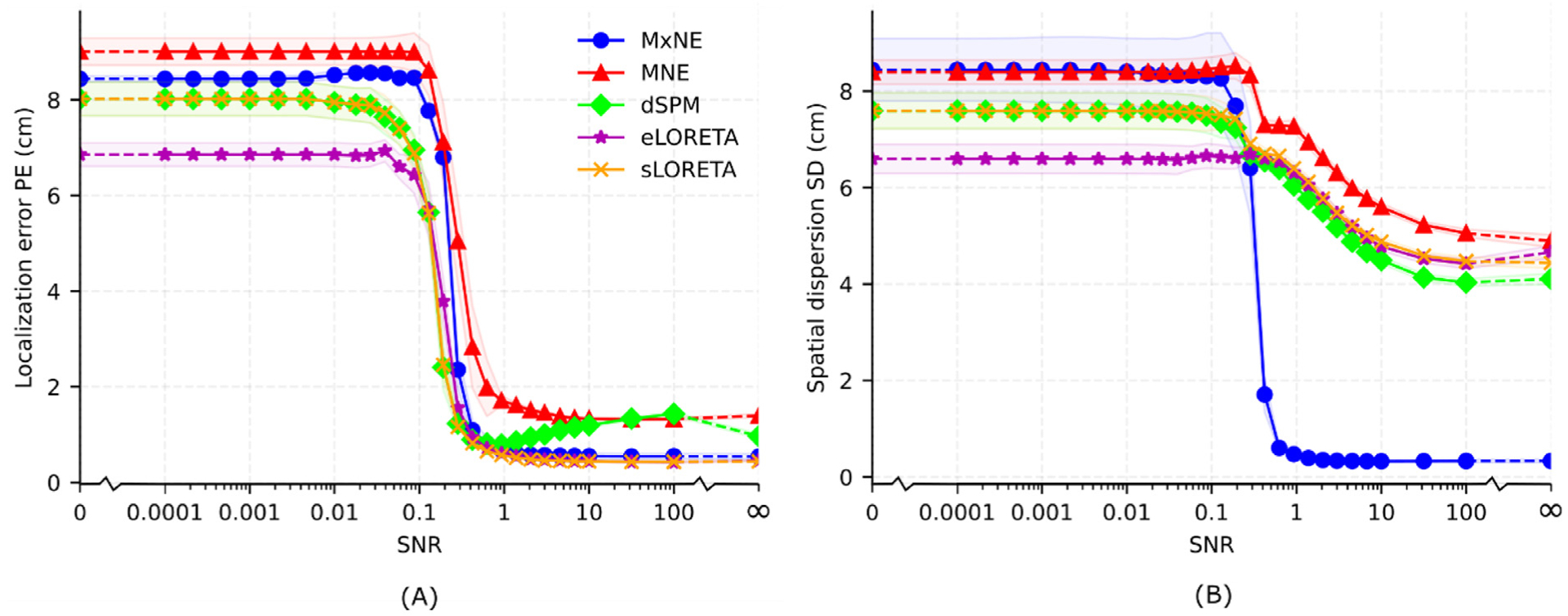
Median localization error PE (A) and spatial dispersion SD (B) over all brain sources as a function of SNR. The solid line represents the median and the transparent region p/m one standard error over subjects. Note that the x-axis is logarithmic but has discontinuities (marked as breaks) as it includes data points with no signal (SNR = 0) and no noise (infinite SNR). The discontinuous segments are dashed.

Fig. 6 shows the median localization error PE and spatial dispersion SD as a function of SNR. Both localization error and spatial dispersion decreases monotonically with SNR for all methods except for a few data points; PE notably increases for dSPM when SNR *>* 1. Apart from these irregularities, SD and particularly PE exhibit largely sigmoidal relationships with SNR. As SNR increases, localization error decreases first and then spatial dispersion; PE decreases from 6–9 cm for SNR = 0.01 (same as random guessing) to 0.5–2 cm for SNR = 1 while SD decreases from 6–9 cm for SNR = 0.1 (same as random guessing) to 4–6 cm for SNR = 10 for the linear methods. Here, random guessing refers to randomizing all estimated source amplitudes between 0 and 1. As was observed in Fig. 5, MxNE has a significantly lower spatial dispersion for higher SNR. While the spatial dispersion of the linear methods decreases continuously for SNR *>* 0.1, the spatial dispersion of MxNE decreases sharply between 0.1 and 1 and then plateaus for SNR *>* 1. Variation across subjects generally decreases as SNR increases, as illustrated by the transparent regions getting smaller as SNR gets higher in Fig. 6.

### 3.3. Classifier performance (ROC/PRC)

**Fig. 7.**
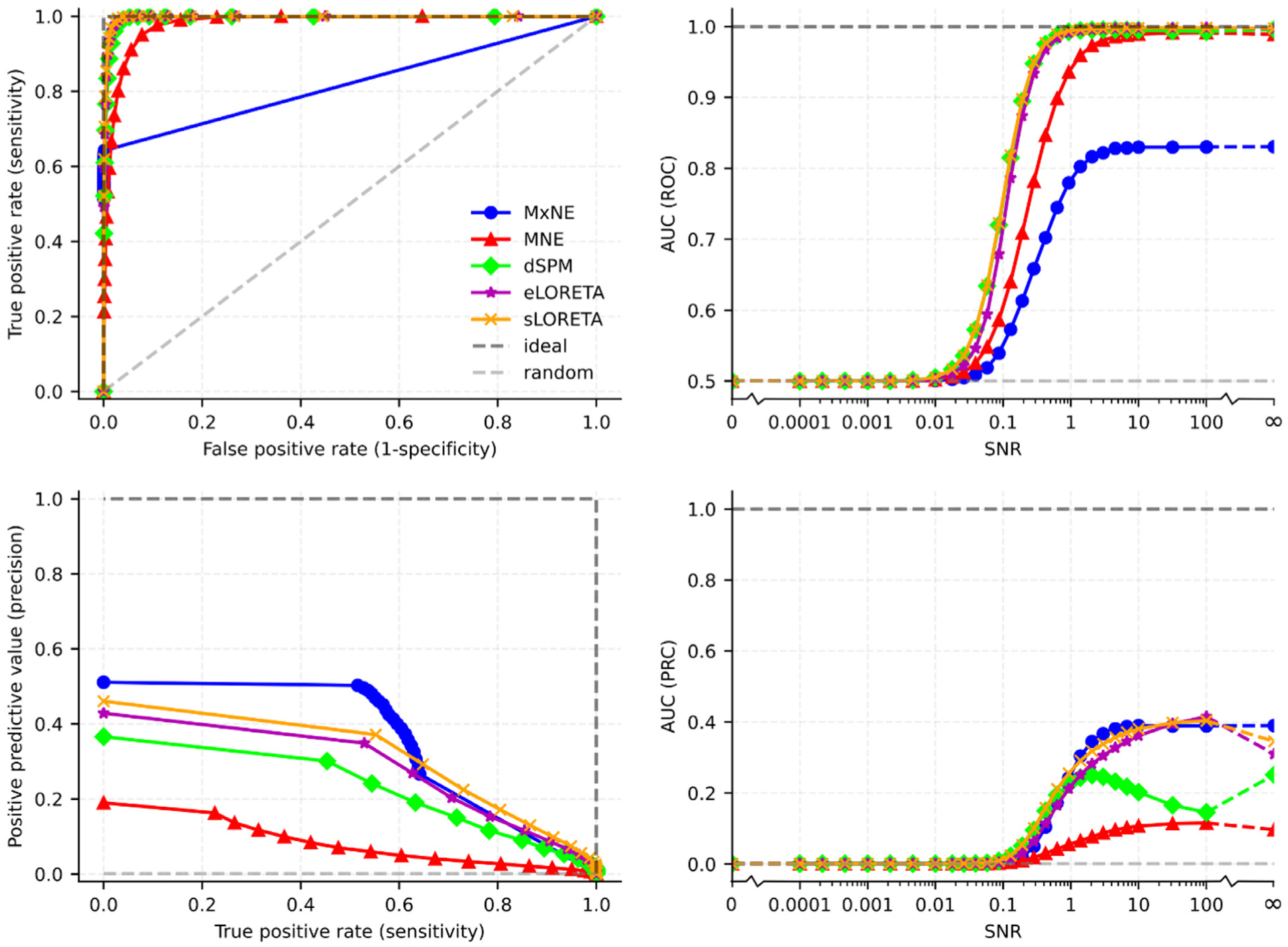
Active/not active source classification results averaged across subjects. Receiver-operator characteristic (ROC) curves are in the upper left, precision-recall curves (PRC) in the lower left and area under the ROC and PRC curves are in the upper and lower right, respectively. The PRC and ROC curves in the left column are for SNR = 3. The light gray dashed line is the expectation value from random guessing and the dark gray dashed line is the result from an ideal classifier. Note that the x-axis in the right column is logarithmic but has discontinuities (marked as breaks) as it includes data points with no signal (SNR = 0) and no noise (infinite SNR). The discontinuous segments are dashed.

The left column of Fig. 7 shows ROC and PRC curves for SNR = 3 and the right column aggregates the classifier performance of these methods in AUC metrics and displays how they vary with SNR. While the sigmoidal relationship between SNR and spatial fidelity as quantified in localization error and spatial dispersion observed in Fig. 6 extends to classifier performance, it is clearer in AUROC than AUPRC. Particularly for dSPM where the AUPRC decreases considerably for SNR *>* 1 because of the increase in localization error that was observed in Fig. 6. Modeling AUROC with

$$\text{AUROC}(\text{SNR})=a\text{ tanh}\left(b{\text{log}}_{10}(\text{SNR})+c\right)+d,$$

where *a*, *b*, *c*, *d* are free parameters, and performing a least squares regression results in a coefficient of determination *r*^2^ *>* 0.8 for all methods. While the AUROC does not differ too much between the linear methods, MxNE performs significantly worse in this framework. However, it performs well as measured in AUPRC. This disparity is due to the lower spatial dispersion of MxNE leading to fewer positives which yields overall lower sensitivity but higher precision. The poor performance of MNE in the AUPRC framework is mainly explained by the relatively high localization error of deeper sources (Fig. 5).

## 4. Discussion

In this study, we expanded the use of the empirical resolution matrix, first introduced in Krishnaswamy et al. (2017), to a generalized evaluation framework that can be used to quantify different aspects of the spatial fidelity of both linear and non-linear estimates for a variety of patch activations, waveforms and signal-to-noise ratios. Using this framework, we emphasized how the spatial fidelity varies as a function of SNR. It was found that there is a largely sigmoidal relationship between SNR and spatial fidelity, quantified here as localization accuracy, spatial dispersion, and AUC. Because of the generality of this framework, many other activation patterns and SNRs could be tested as well, which could be useful in source estimation analysis and evaluation.

### 4.1. Comparison to earlier studies

The MNE and dSPM peak localization errors presented in Figs. 5 and 6 agree roughly with those reported in previous studies using the noiseless closed-form resolution matrix from point sources; Hauk et al. (2011) and Hauk et al. (2019) reported peak localization errors to be in the range of 0 – 5 cm using MNE and dSPM. The localization errors found in this study using dSPM were somewhat smaller than those reported by Molins et al. (2008); 90% of all localization errors were smaller than 2 cm for SNR = 3 using dSPM in this study, compared to 3.5 cm for noiseless estimates in Molins et al. (2008). Our results on spatial dispersion are higher than those reported in Hauk et al. (2019); Hauk et al. (2011); Molins et al. (2008). However, the topographies of these metrics as shown in Fig. 5 are similar to those found in previous studies. The discrepancies are partly due to the patch activations (instead of point sources as in earlier studies) and partly due to our measure of spatial dispersion as the weighed *ℓ*_1_ norm of the distances between sources instead of the weighted *ℓ*_2_ norm that was used in the previous studies. When applying the noiseless closed-form resolution matrix for point sources and using the same formulae for spatial dispersion, we could reproduce their results except for the spatial dispersions reported by Molins et al. (2008); this comparison is shown in the Appendix.

It is well-known that s/eLORETA have zero localization error in the whole brain for single point sources, since the inverse kernel is scaled to explicitly guarantee that the location of the peak reconstruction amplitude coincides with that of the active source (Hauk et al., 2011; Pascual-Marqui, 2002; Pascual-Marqui et al., 2011). This was also verified in the present study when using the analytical resolution matrix and point sources as the source model. As was shown here, however, this zero localization error only holds true for point sources and noiseless sensor data (Figs. 5 and 6). Since patch activation is a more physiologically accurate source model than point sources (Hämäläinen et al., 1993; Murakami and Okada, 2015; Okada, 1989), and sensor data are never completely noiseless, it can be concluded that the idea of zero localization error in sLORETA and eLORETA is largely an academic concept although still a desirable property in an estimator which does lead to smaller localization errors even for noisy data (Figs. 5 and 6).

Attal and Schwartz (2013) performed a simulation study of localization of subcortical activity in the amygdala, hippocampus, and thalamus with MEG. In that study, an energy SNR of 20 was assumed which implies an amplitude SNR of about 4.5. The reported localization error was on the order of 1 – 2 cm with dSPM and sLORETA, which is in rough agreement with the present study’s results on mislocalization in the medial cortical area around the mentioned subcortical structures.

### 4.2. Relationship between the ROC/PRC framework and resolution metrics

Both the AUPRC and AUROC had a largely sigmoidal relationship to SNR (Fig. 7), much like the other resolution metrics. In the case of AUROC, the increase with SNR was mainly explained by TPR rather than FPR because FPR was always relatively low, even for high noise levels. As SNR increased, FPR decreased further but the relative change was small and therefore only mildly influenced AUROC while the relative increase in TPR was significant. As has been shown before, e.g., by Saito and Rehmsmeier (2015), AUPRC could be a more informative evaluation metric than AUROC for imbalanced data sets such as the one in the present study where we have 999000 negatives and 1000 positives. This is because the precision of the classifier can be low, i.e., a low share of true positives among all data points classified as positives, and still yield a very high AUROC because the denominator of FPR is dominated by the overwhelming amount of true negatives. This is indeed the case in the estimators examined here; while the AUROC are driven high by a relatively low FPR caused by a large amount of true negatives, the AUPRC shows that the precision of the estimators are relatively poor, i.e., of all sources estimated to be active, the majority are false even for high thresholds and SNR. This is particularly true for estimators with a higher localization error (dSPM and MNE) and less so for s/eLORETA and the mixed-norm approach. It is noteworthy that even for infinite SNR, highest possible threshold and using MxNE, which had the highest precision of all estimators, about half of all sources (patches) that were estimated as active were false positives. Because this is the best-case scenario, i.e., lower SNR, another source estimation method, lower threshold or more active sources, should all give an even lower precision, the share of false positives among all sources estimated as active in real evoked response studies is likely much higher than 50 %.

The non-linear MxNE method performed poorly in the ROC framework, despite low localization error and very low spatial dispersion (Figs. 5, 6), which both generally correlated negatively with AUC. However, it performed better in the PRC framework. The reason for this seemingly paradoxical performance lies in the sparsity of the source reconstruction. Even though the localization error with MxNE was low, it was not zero (Fig. 5) and because of the extremely low degree of spatial dispersion, MxNE estimated activity in the active patch to be zero in 34% of all cases even when SNR was infinite. This means that the sensitivity was never above 66% even when the discrimination threshold value *T* was near zero, causing the poor performance as quantified in AUROC. Because the source reconstruction amplitudes of most sources were estimated as zero with MxNE, the FPR was also very low when *T* approached zero which explains the characteristic ROC curve for MxNE in Fig. 7; the ROC went directly from a low TPR and FPR to (FPR, TPR) = (1,1) when the threshold was set to zero and all sources were considered active. Although the low spatial dispersion caused a poor performance as quantified in AUROC, it led to a good performance in AUPRC because of the high precision; a lower spatial dispersion leads to fewer false positives. Whether AUPRC or AUROC is a better metric for estimator performance depends on the question asked, which is discussed in the next subsection.

### 4.3. Spatial fidelity comparison between source estimation methods

The linear distributed source estimation methods tested here, MNE, dSPM, sLORETA, and eLORETA, performed overall about equally well, although dSPM had higher localization error for higher SNR and MNE had slightly higher localization error and spatial dispersion (Fig. 6), leading to somewhat worse performance in the aggregate AUC frameworks (Fig. 7). MxNE, sLORETA and eLORETA had lower localization error than dSPM and MNE for SNR *>* 0.1, PE being around 4–6 mm. MxNE had significantly lower spatial dispersion (Fig. 6). The spatial dispersion with MxNE was, however, often smaller than the localization error (Fig. 6), leading to the active source being estimated to zero in 34% of all cases, even for high SNR (Fig. 7), causing low sensitivity and therefore poor performance in the aggregate performance metric AUROC. However, the low spatial dispersion also caused a much lower number of false positives and therefore higher precision and better performance as quantified in AUPRC. sLORETA performed overall well, having one of the highest AUC (ROC/PRC) and lowest localization error for all SNR. Its performance was the same as that of dSPM when SNR *<* 0.1 but diverged for SNR *>* 0.1 when the performance gain of dSPM with increasing SNR slows down and eventually starts reversing, since sLORETA and dSPM converge to the same estimate when the Tikhonov regularization parameter *λ* (Eq. 4) gets much larger than unity, which happens for low SNR since *λ* = SN R^−1^. This result highlights the limitations of only normalizing the estimates with respect to the noise covariance as in dSPM instead of including the signal as well, as in sLORETA, when SNR is high.

The outlier in terms of estimator performance was the non-linear MxNE which had significantly lower spatial dispersion because of the sparse source reconstruction. The lower spatial dispersion caused lower sensitivity but higher precision. In the end, no method was objectively better than all others in every aspect but instead the appropriate method to use depends on the scientific question being asked and the context of the problem. For example, in a cross-modal study one might need to assess the neural current density in physical dimensions rather than statistical entities where non-standardized MNE estimates could prove more useful. Or in a deductive hypothesis testing study, a low rate of false positives (high precision) could be more important than high sensitivity, especially in functional neuroimaging which as a research field is relatively prone to false positive findings, while the opposite could be true for clinical studies or inductive observation studies. The situation is further complicated by the introduction of many sources, e.g., functional connectivity studies, which we did not consider in this study.

### 4.4. Influence of SNR: Implications for experiments

Taken together, the performance of the source estimates tested here are not significantly better than random guessing for SNR *<* 0.01, then improves rapidly between 0.01 *<* SNR *<* 1 and slightly above SNR *>* 3. The source estimates then improve only marginally above SNR *>* 10. This information could be useful when assessing how many epochs are needed to adequately map evoked potentials in evoked response experiments and at which point there is no longer any added value of increasing SNR further. This information could also be interesting for the emerging field of subcortical M/EEG, where the larger distances between the sources and sensors cause a lower SNR than in cortical M/EEG which has caused controversy on whether or not it is possible to detect and adequately reconstruct sources in the subcortex (Attal and Schwartz, 2013; Krishnaswamy et al., 2017; Samuelsson et al., 2019; Samuelsson et al., 2020; Seeber et al., 2019).

The fact that the spatial dispersion converged to around 4 – 5 cm and localization error to 0.5 – 1.5 cm for the linear methods even when SNR goes to infinity showcases how the challenge of the inverse problem is not only due to the relatively low SNR in M/EEG recordings but also due to the ill-posedness of the inverse problem. These observations demonstrate the influence of SNR on spatial fidelity in M/EEG source estimation and the importance of including noise when evaluating source estimates.

### 4.5. Methodological considerations

The approach and analytical tools presented in this study is generally applicable to all distributed source estimates using any MEG or EEG system. The results in this study, however, are based on the application of this general approach specifically to the Elekta-Neuromag VectorView MEG with a 70-channel MEG-compatible EasyCap EEG. A different MEG system or EEG cap of different channel density could give a different result. Using MEG or EEG separately would probably give worse results than those presented here, based on previous studies (Liu et al., 2002; Molins et al., 2008).

The analytical resolution matrix is considerably easier to compute than the empirical resolution matrix presented in this study; while the analytical resolution matrix takes on the order of a few seconds to compute, the time cost of the empirical resolution matrix depends on many factors such as the duration of the waveform and the time cost of the inverse method. In the implementation used in the present study, it takes about five minutes to compute the MNE empirical resolution matrix in one subject using a single processing thread on a modern desktop computer, although the code has support for parallelization. In terms of the source space, the analytical resolution matrix approach is also more general since it is not bound to a set of pre-defined patch activations. Moreover, the noise term *N*, here taken as resting state data, is dominated by background brain activity which is explainable by the forward model and is not considered noise but signal in some cases e.g., resting state connectivity analysis. For distributed source methods in particular which aim to explain the recorded data with neural currents within the source space, one could argue that all data that seem to originate from the source space, such as background brain activity, should not be considered noise but signal and the only logical choice for the noise term is then empty room data. Furthermore, one can get some insight into estimators’ robustness to noise by varying the Tikhonov regularization parameter. Although one should take note that even when the Tikhonov regularization parameter is perfectly tuned as in the present study where the SNR was predetermined, the estimates got substantially worse for SNR *<* 10, and especially when SNR decreased below unity, showcasing that even when the Tikhonov parameter is ideal, there are limits to the regularization’s effect on noise mitigation. Nonetheless, the analytical resolution matrix does have several advantages over the empirical resolution matrix, and the analytical framework presented in this study is not meant as a replacement to the analytical resolution matrix but rather as a complement since it enables investigation of some aspects that is not possible or at least non-trivial to assess with the analytical resolution matrix such as robustness to SNR and comparison between linear and non-linear estimators.

The spatial fidelity of M/EEG source estimates depends on many factors. In this study we particularly emphasized the influence of SNR and choice of estimator. Other factors that could influence these results include the number of sensors, the number of simultaneously active sources, and the degree of correlation between the active sources, as well as errors in the head-MRI-MEG co-registration. The performance of contextual methods such as TF-MxNE, Kalman filter approaches or C-MNE also depend on the activation waveforms (Dinh et al., 2019; Gramfort et al., 2013b; Lamus et al., 2012). Testing and comparing the spatial resolution of contextual methods to non-contextual methods for different activation waveforms using the approach presented here could be a topic of a future study.

In the classifier performance assessment presented here, there was exactly one active source at any given time and reconstructed activity at this source was the only reconstruction classified as a true positive. One could also consider any source within that brain region to be a correct reconstruction. In the strict classification framework used here, a mislocalization of less than a centimeter is classified as a false positive. If a looser definition were to be implemented, all methods would have performed better but particularly MxNE since it often estimated the active patch to be zero due to the very low spatial dispersion, but had a median localization error of less than one centimeter for high SNR (Fig. 6).

A common way of quantifying localization accuracy for distributed source estimates is by the center of gravity error *E*_*cg*_,

$$E_{cg}={\left\|r_{i}-\frac{\sum_{j=1}^{N_{s}}\left|{\hat{x}}_{j}\right|r_{j}}{\sum_{j=1}^{N_{s}}\left|{\hat{x}}_{j}\right|}\right\|}_{2},$$

where ***r***_*i*_ is the location of the active source *i*. This is, however, an inadequate quantification of localization accuracy when employing a cortical surface source space because the center of gravity has a bias towards the center of the brain. This happens because all energy in the source estimate is constrained to the cortex; a uniform source estimate around an activated deep source results in the center of gravity coinciding with the activation point due to the symmetry in the cortex around the deep source, but not in the superficial case because then a uniform source estimate results in a center of gravity inside the cortex, away from the activated source point. Therefore, the anterior and posterior cortical areas of the brain will appear to exhibit particularly high localization errors while the deep, central regions of the brain will appear to demonstrate low localization errors, which is the inverse of what you would expect from inverse methods with a cortical bias such as MNE. The peak reconstruction error that was used in this study to quantify localization error is a common measure of mislocalization. However, it does not account for distance over the cortical manifold, e.g., reconstructing a source at the opposite wall of a sulci results in a very small peak reconstruction error but large distance over the cortical manifold that could belong to a different functional region.

### 4.6. Generalizability to multiple active sources

In the present study we only considered one active source at any one time. During many evoked responses, and in brain activity in general, there are many sources that are simultaneously active. The concept of the empirical resolution matrix could be extended to many simultaneously active sources simply by activating more than one patch at a time or, if the methods are linear, sum different columns because of the superposition principle, although one would need to scale the noise term accordingly.

However, it would be difficult to assess all possible active source combinations by multiple patch activations, since for *N* active patches, there would be $\left(\begin{array}{c} 1000\\ N \end{array}\right)=\frac{1000!}{N!(1000-N)!}$ possible configurations to test, e.g., 499500 for *N* = 2 patches, 166167000 for *N* = 3 patches etc. Instead one would likely need to resort to Monte Carlo simulations. The resolution metrics PE and SD presented here would not make sense for multiple active sources but would need to be redefined. The classifier ROC/PRC framework could still be used in its present form, however, although the numerical implementation would be different since there would no longer be a one-to-one relationship between active/non-active diagonal/off-diagonal element.

Generally, a lower spatial dispersion means better ability to distinguish multiple sources. It is therefore tempting to make the conclusion that MxNE would perform better for multiple active sources due to its lower spatial dispersion. However, while this would be true for linear methods due to the superposition principle, this is too hasty of a conclusion to make because of the non-linearity of MxNE; the estimate resulting from two or more active sources is generally not the same as the sum of the estimates resulting from these sources being individually active. While it is possible that the lower spatial dispersion does make MxNE better at distinguishing multiple active sources, it is not guaranteed.

## 5. Conclusion

A general approach and analytical tools for quantifying the spatial fidelity of MEG/EEG source estimates were presented. These tools allow for evaluation of both linear and non-linear inverse solvers for different activation waveforms and SNR, which is critical since SNR in MEG/EEG recordings is typically moderate to low. It was found that spatial fidelity quantified in terms of localization error, spatial dispersion and the aggregate AUC metrics varies significantly with SNR, following a largely sigmoidal relationship. As a rule of thumb, SNR should be at least 0.1 for inverse modeling to be relevant and 3 to get good estimates, while increasing SNR over 10 results in little added benefit. The spatial fidelity of the linear methods MNE, dSPM, sLORETA and eLORETA was comparable while the non-linear MxNE method had significantly lower spatial dispersion causing a lower sensitivity but higher precision than the linear methods in most settings. We believe that the results and tools presented here will be useful for researchers in source estimate analysis and for methods developers since this provides a general and objective evaluation framework.

## Figures and Tables

**Fig. 8.**
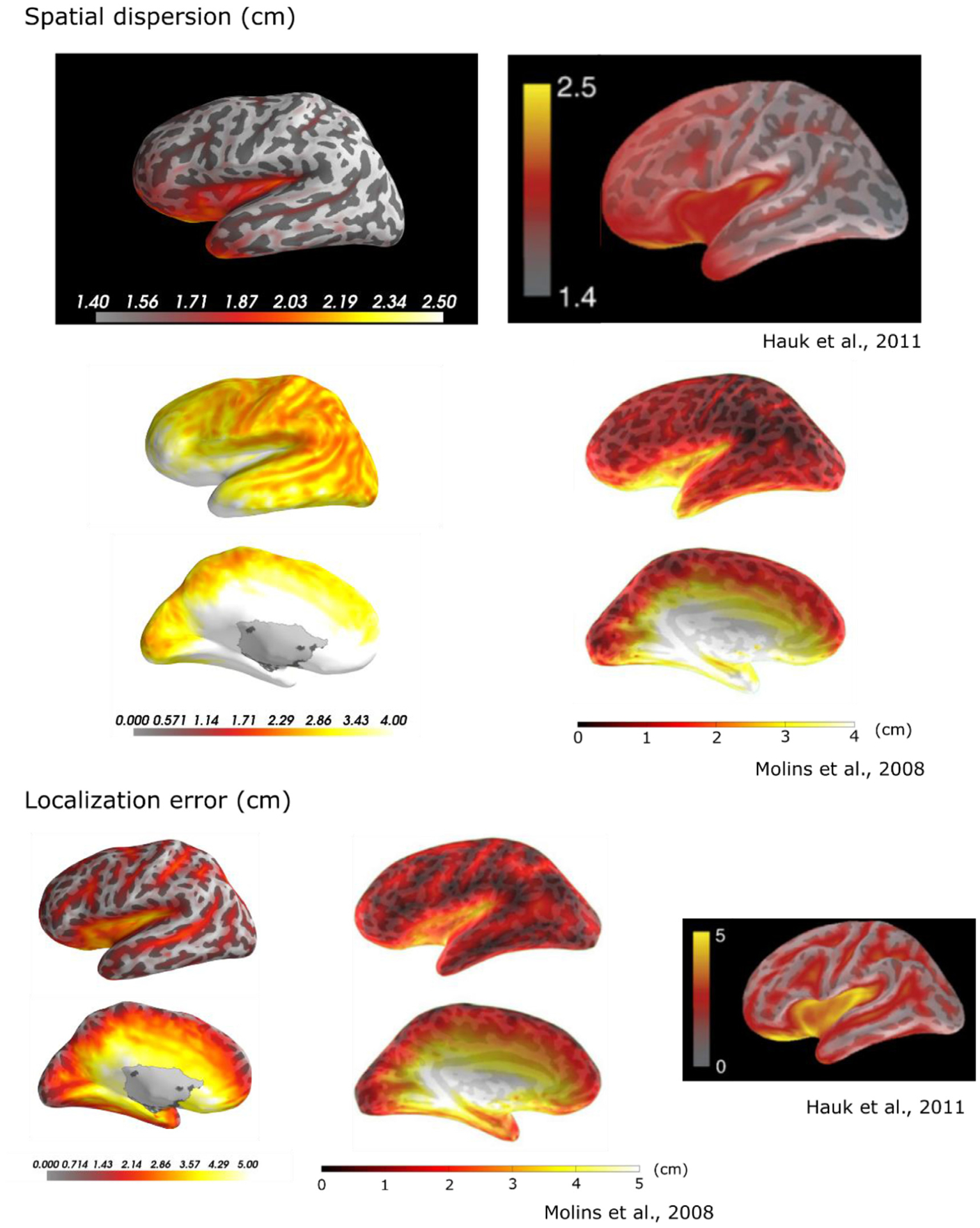
Comparison to previous studies. The same metrics for spatial dispersion and localization error as were used in Hauk et al. (2011) and Molins et al. (2008) were computed in one subject using the analytical resolution matrix and dSPM. The left column shows data from the present study. Note that the unit for spatial dispersion in Hauk et al. (2011) is $\sqrt{\text{cm}}$, and that the data from Hauk et al. (2011) assumes only MEG data while the present study and Molins et al. (2008) assumes both MEG and EEG data.

## Appendix

Fig. 8 shows a comparison with previous studies. Metrics for spatial dispersion were recomputed using the formulae in Hauk et al. (2011) and Molins et al. (2008), respectively, while the formula for localization error was the same in all studies. The analytical resolution matrix and dSPM were used in order to be consistent with the previous studies. Note that the results from Hauk et al. (2011) were derived only from MEG data, so we should expect slightly higher spatial dispersion and localization error than in the present study and Molins et al. (2008) who both presumed combined M/EEG recordings. This is indeed the case, as Fig. 8 shows; the overall spatial structures of the spatial dispersion and localization error are similar to those in the present study but just slightly higher (because we use different subject data we should not expect a perfect match). The reported localization error was also largely consistent across the present study and Molins et al. (2008), but the spatial dispersion was conflicting, being lower in Molins et al. (2008). It is possible that this discrepancy is due to a computing error in Molins et al. (2008), since the reported spatial dispersion being close to zero in the lateral cortical mantle is extremely low, even for noise free data.
